# First Known Feeding Trace of the Eocene Bottom-Dwelling Fish *Notogoneus osculus* and Its Paleontological Significance

**DOI:** 10.1371/journal.pone.0010420

**Published:** 2010-05-05

**Authors:** Anthony J. Martin, Gonzalo M. Vazquez-Prokopec, Michael Page

**Affiliations:** Department of Environmental Studies, Emory University, Atlanta, Georgia, United States of America; Raymond M. Alf Museum of Paleontology, United States of America

## Abstract

**Background:**

The Green River Formation (early Eocene, about 42–53 *Ma*) at and near Fossil Butte National Monument in Wyoming, USA, is world famous for its exquisitely preserved freshwater teleost fish in the former Fossil Lake. Nonetheless, trace fossils attributed to fish interacting with the lake bottom are apparently rare, and have not been associated directly with any fish species. Here we interpret the first known feeding and swimming trace fossil of the teleost *Notogoneus osculus* Cope (Teleostei: Gonorynchidae), which is also represented as a body fossil in the same stratum.

**Methodology/Principal Findings:**

A standard description of the trace fossil, identified as *Undichna* cf. *U. simplicatas*, was augmented by high-resolution digital images and spatial and mathematical analyses, which allowed for detailed interpretations of the anatomy, swimming mode, feeding behavior, and body size of the tracemaker. Our analysis indicates that the tracemaker was about 45 cm long; used its caudal, anal, and pelvic fins (the posterior half of its body) to make the swimming traces; and used a ventrally oriented mouth to make overlapping feeding marks. We hypothesize that the tracemaker was an adult *Notogoneus osculus*.

**Conclusions/Significance:**

Our results are the first to link a specific teleost tracemaker with a trace fossil from the Green River Formation, while also interpreting the size and relative age of the tracemaker. The normal feeding and swimming behaviors indicated by the trace fossil indicate temporarily oxygenated benthic conditions in the deepest part of Fossil Lake, counter to most paleoecological interpretations of this deposit. Lastly, our spatial and mathematical analyses significantly update and advance previous approaches to the study of teleost trace fossils.

## Introduction

The Green River Formation (early Eocene, about 42–53 *Ma*), a semitropical lacustrine deposit in the western U.S., is world-famous for its exquisitely preserved fossil-fish assemblage, particularly in the area of Fossil Butte National Monument in Wyoming, USA [1], [2], [3], [4], [5]. Fossil Lake, located in Fossil Butte National Monument, is the smallest in area yet is interpreted as the deepest of several lakes in the region during the late Paleocene through the middle Eocene [4], [6], [8]. Thus far, 23 species of fish are identified from Fossil Lake strata.

One of these fish species, *Notogoneus osculus* Cope (Teleostei: Gonorynchidae), is the only one restricted to a single fossiliferous bed (F-1 of 1), colloquially called the “18-inch Layer.” *N. osculus*, the type species for the genus, is also notable for its ventrally oriented mouthparts, a rare anatomical trait among Green River Formation teleosts, supporting its interpretation as a bottom-feeder [1], [5]. Nonetheless, no other fossil evidence has supported this diagnosis, and bottom feeding by *N. osculus* in Fossil Lake is at odds with some sedimentological and geochemical interpretations for the 18-inch Layer, interpreted as the deepest part of the lake and hypothesized as anoxic, dysaerobic, or otherwise hostile to benthic fauna [1], [3], [4], [9]. Indeed, well-preserved teleost body fossils in the 18-inch Layer and other similar Green River strata, along with little evidence of bioturbation and preservation of kerogen-rich layers, were often cited as evidence for anoxic conditions [1], [3], [10]. On the other hand, body fossils of bottom-feeding fish, such as *N. osculus*, a catfish (*Astephus antiquus*), and rays in Fossil Lake suggest that bottom waters were occasionally aerobic enough to allow for these fish in deeper parts of the lake. Moreover, some seasonal mixing is suggested by alternation of kerogen-rich layers with micrite [2], [4].

As a result, the discovery of an extraordinary trace fossil from the 18-inch Layer, which we attribute to *N. osculus*, lends new insights on its behavior, as well as the paleoecology of Fossil Lake. The trace fossil (FOBU-12718) indicates swimming and systematic benthic feeding by a teleost with downward-pointing mouthparts, linking it anatomically with *N. osculus*. Moreover, our calculations of dimensions and other aspects of the trace fossil are anatomically consistent with a adult tracemaker, based on recent growth series defined for this species [5]. Lastly, and perhaps most importantly, this trace fossil, along with several other teleost swimming traces from the same stratum, demonstrate normal swimming on the sediment-water interface in the deepest part of Fossil Lake. This evidence thus supports the probability of occasional oxygenation of Fossil Lake bottom waters in its deepest area, and accordingly refutes assumptions that near-benthic fish were permanently excluded from this paleoecosystem by anoxia, thermoclines, or other ecological factors [1], [3]. As a result, the paleoecology of Fossil Lake is now better understood to include the role of teleost bottom feeding as a part of nutrient cycling in its deep-water benthic communities.

## Methods

### Background: Locality, Stratigraphy, Lithology

**Figure 1:**
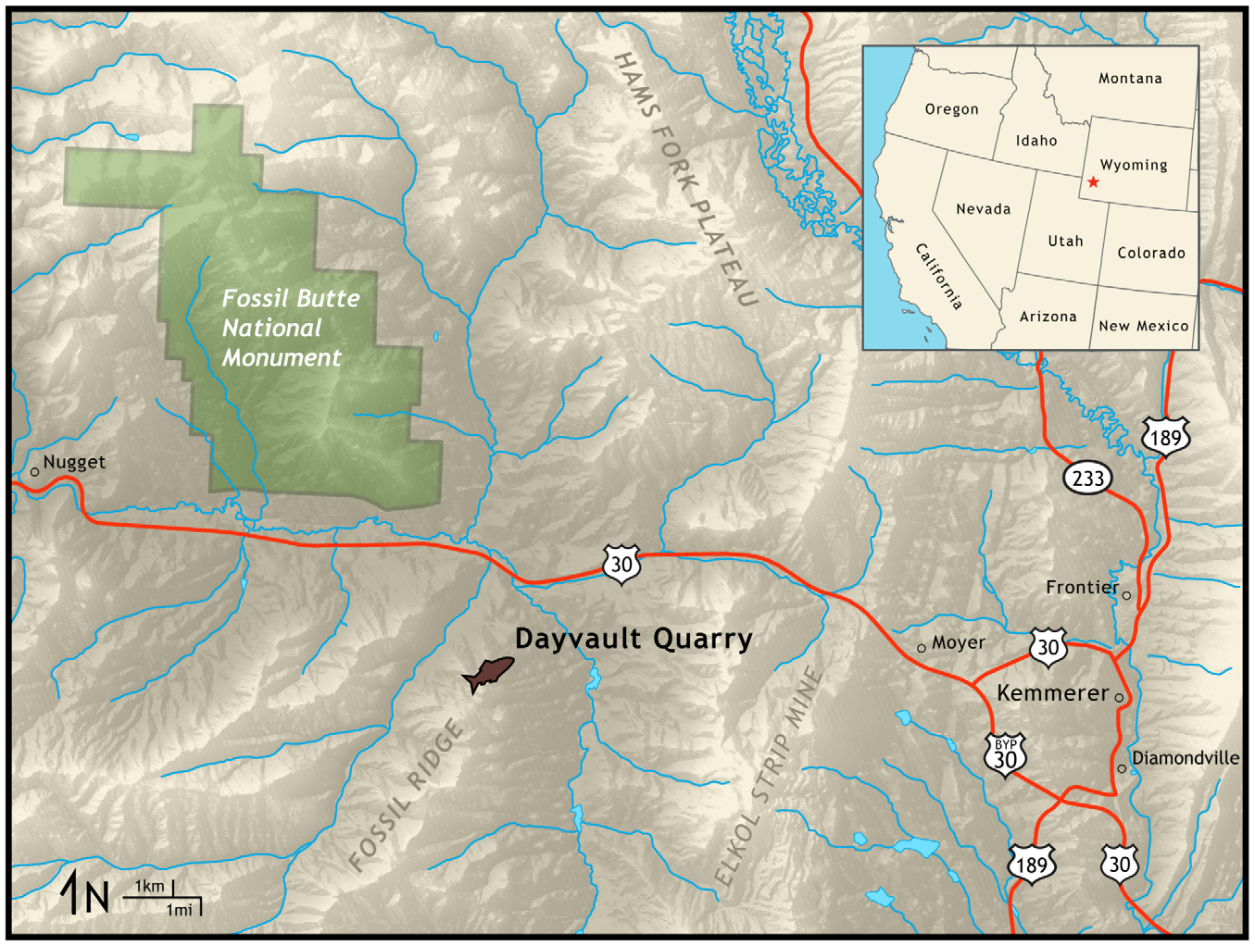
Locality map of Dayvalult Quarry, source of specimen FOBU-12718, with relation to Fossil Butte National Monument, Wyoming (USA).

FOBU-12718 was recovered from the Dayvault Quarry, which is adjacent to Fossil Butte National Monument and on Wyoming public land (Figure 1). This quarry is privately leased to qualified fossil collectors, and one of these collectors (Warfield Fossils, Inc.) donated the specimen to Fossil Butte National Monument. The specimen comes from the 18-inch Layer, which is in the Middle Unit of the Fossil Butte Member, Green River Formation. A potassium-feldspar tuff at the top of the Middle Unit was originally dated via ^40^K/^40^Ar as 50.2±1.9 *Ma* [4]. More recently, the same tuff bed was dated again through ^40^K/^40^Ar, resulting in a weighted mean age of 51.66±0.09 *Ma* [8]. Regardless of exact dating, the 18-inch layer is slightly older than these dates, and falls into the latest part of the early Eocene, well within the Ypresian Age (55.8 to 48.6 *Ma*) or Wasatchian North American Land Mammal Age (about 55-51 *Ma*). The 18-inch Layer is well known for its exquisitely preserved fossil fish, particularly teleosts, but also includes body fossils of plants, insects, birds, mammals, and reptiles [1], [2], [4]. The host lithology is a very light gray to yellowish gray (N8 to 5Y 8/1, respectively, on the Munsell color range), kerogen-rich laminated micrite (or KRLM, *sensu* [3]), with less than 5% clastic content [1]. Organic carbon contents in KRLMs of the Middle Unit are 2–14% [3], [4].

The trace fossil is discernable via exposure of darker, pale to moderate yellowish brown (10YR 6/2 to 5/4), kerogen-rich mud in a lamina just below the lighter-colored micritic surface, as well as through slight variations in relief along the planar surface. FOBU-12718 is from a horizon about 12.5 cm below the top of the 18-inch Layer, and consists of a part (FOBU-12718A) and counterpart (FOBU-12718B). Unless indicated otherwise, descriptions are of the part, which preserves the trace fossil in negative relief. The slab containing the studied specimen had been cut into a rectangle, 23×108 cm, for ease of extraction and storage, and the trace fossil is in the central lengthwise portion.

### General Description of Trace Fossil

**Figure 2:**
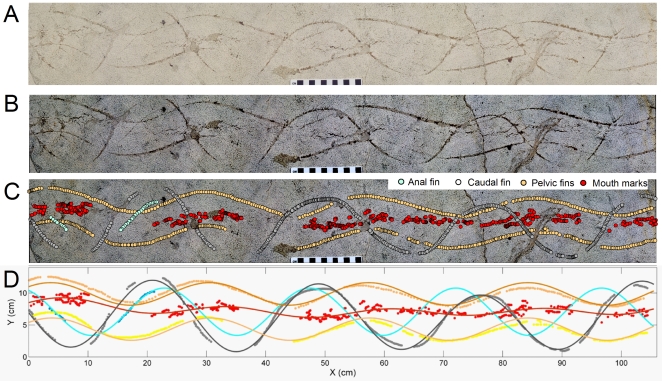
Trace fossil specimen FOBU-12718. A - Digital composite photograph of specimen. B – Digitally enhanced composite photograph, emphasizing contrast of trace fossil from host lithology. C – Digitized points assigned to waveforms in the trace fossil, with labeling tentatively assigned to presumed body parts (caudal fin, anal fin, pelvic fins, mouth). D – Fitted waveforms based on Fourier transform, showing extrapolated paths of body parts, and superimposed onto plots taken from trace fossil. Colors of fitted waveforms describe each presumed body part, as indicated in the legend.

The trace fossil contains several interrelated waveforms (Figure 2A and Text S1). Among these are thin (<2 mm wide), shallowly impressed (<1 mm depth), paired, parallel, and synchronous grooves, separated by 5.2–5.5 cm and forming discontinuous sine-like waveforms of relatively low amplitude (3–4 cm) and long wavelength (27–28 cm). Three complete and two partial wavelengths of these coupled traces are preserved along the length of the slab. These trails are cross-cut by a single discontinuous waveform with a higher amplitude (9–10 cm), although its 27–28 cm wavelength is identical to those of the paired waveforms. This single waveform consists of four complete cycles and two partial ones. Another single but short (10 cm long), discontinuous segment of another waveform is slightly offset (2.8 cm maximum distance) from the high-amplitude one; its amplitude is less than that of the high-amplitude waveform. Medial to the paired and parallel waveforms are discontinuous markings consisting of incomplete, overlapping ellipsoids, about 1–2 cm wide, which join and bifurcate in places. (Please refer to Figure 2 and Text S1 for a detailed view of these waveforms.) The trend of these traces, however, is not strictly medial, and is occasionally proximal to either of the paired trails along the length of the trace fossil. Where these traces approach one side, the opposite-side parallel waveform is thinner or not recorded, resulting in consistent and predictable gaps in each waveform.

Thin, shallow impressions forming regular waveforms are assignable as trace fossils to the ichnogenus *Undichna* [11]; moreover, paired, in-phase waveforms in association with a higher-amplitude waveform are best identified as *Undichna* cf. *simplicitas* [12], [13]. Hence we identify FOBU-12718 as *Undichna* cf. *simplicitas*, and it is preliminarily interpreted as a trace made by a swimming teleost fish.

### Spatial Analysis of Trace Fossil

In order to better quantify the motion and size of the tracemaker, we conducted a spatial analysis of FOBU-12718. An actual-sized color digital composite photograph of FOBU-12718 (Figure 2A) was made through stitching a series of high-resolution digital photographs using Adobe Photoshop™ (version CS3). The resulting image was spatially referenced with X-Y coordinates (in mm) using ArcGIS 9.2 software (ESRI, Redlands, Colorado). Contrast between the traces and the surrounding sediment was further enhanced by applying a Principal Component (PCA) filter [14] and representing the first component as a negative grayscale image (Figure 2B). Visually identifiable traces were then digitized from the enhanced image (Figure 2C) and the X-Y coordinates saved as a separate table using ArcGIS 9.2. In our support of open-access scientific research, we have made the raw data available as a supplementary file (File S1). Resultant X-Y coordinates for each trace were incorporated in the curve-fitting toolbox of Matlab 9.1 (Mathworks, Natick, Massachusetts). For the sake of the analysis, each waveform was preliminarily assigned to its putative appendages (e.g., pelvic, caudal fins).

A harmonic analysis was performed to describe the mathematical properties of the traces. By fitting the digitized data to a Fourier series [15], we described the wavelength and amplitude of each waveform. We also extrapolated the path taken by each body part of the tracemaker that did not interact directly with the sedimentary surface. With this analytical procedure, we sought to estimate the lateral amplitude of every part of the tracemaker body from anterior to posterior, and ultimately the size of the tracemaker. Mathematically, a Fourier series with period *j* (*f_j_*) can be represented as a simple combination of sine and cosine functions as follows:
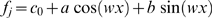
with Fourier coefficients c_0_, a_n_ and b_n_ defined by the integrals:
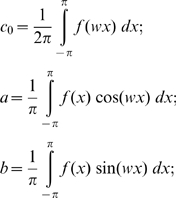



From the above formula and coefficients, and considering that measurements of FOBU-12718 were made in Euclidian (XY) space, we were able to estimate the frequency (i.e., distance between two successive wave peaks) and maximum amplitude (i.e., distance between high and low wave) of the waves associated with each putative appendage. Briefly, the wave frequency of an appendage *j* (λ_j_) in Euclidian space was derived by the formulae λ_j_ = (*2π/w_j_*), whereas the 95% confidence intervals (CI) were derived by applying the same formulae to the CI of *w*_j_. Similarly, the maximum wave amplitude and CI of appendage *j* (*A_j_*) were derived by the formulae *A*_j_ = 2*(a^2^+b^2^)^1/2^, obtained after transforming *f*_j_ to a sum of sine functions (not shown). We applied *f*_j_, λ_j_ and *A_j_* to each appendage's fitted wave coefficients. For the central, overlapping ellipsoidal impressions, a Fourier series was also fitted to estimate the central axis of the digitized traces.

The cyclical changes imparted by the tracemaker body parts, evidenced by impressions in the sediment, formed a running wave that was fitted accurately by a simple Fourier series (Figure 2D, Table 1). The estimated wavelength of this running wave was similar across traces (range, 24.6–27.3 cm: Table 1). The amplitude, however, showed a marked difference between traces (Δ “caudal-mouth” = 8.5 cm, Table 1). Based on these fitted waveforms, we were also able to derive morphometric parameters of the tracemaker. For example, the maximum width of the tracemaker, estimated by calculating the distance between left-right paired impressions, was 5.51 cm, with a range of measurements of 5.4–5.6 cm, whereas the average distance between caudal and pelvic fins was 20.65 cm, with a range of 19.1–22.6 cm (Figure S1). Refer to Text S1 for a detailed description of the methods employed to derive such a distance.

**Table 1:** Model fit and parameter estimates for each waveform associated with traces in FOBU-12718.

	Model fit		Mean fitted parameters (95%CI)			Model estimates (95% CI) in cm	
Track	Adjusted R^2^	RMSE^1^	a	b	w	Wavelength, λ_j_	Maximum amplitude^2^, A_j_
Caudal fin	0.931	0.85	0.90 (0.67–1.14)	−4.80 (−4.94–−4.66)	0.230 (0.229–0.231)	27.3 (27.2–27.4)	9.7 (9.41–10.13)
Anal fin	0.993	0.14	3.33 (3.08–3.58)	−1.61 (−2.21–−1.02)	0.255 (0.240–0.271)	24.6 (23.2–26.2)	7.4 (6.48–8.41)
Pelvic fin (left)	0.835	0.57	1.14 (0.99–1.29)	1.34 (1.20–1.48)	0.234 (0.232–0.236)	26.9 (26.6–27.1)	3.5 (3.11–3.92)
Pelvic fin (right)	0.868	0.49	1.05 (0.91–1.20)	1.39 (1.28–1.50)	0.235 (0.233–0.236)	26.7 (26.6–27.0)	3.5 (3.14–3.84)
Mouth marks	0.402	0.52	0.03 (−0.28–0.20)	0.60 (0.53–0.67)	0.232 (0.227–0.239)	27.1 (26.3–27.7)	1.2 (1.13–1.45)

1 Root Mean Standard Error.

2 Distance between upper and lower peaks of a wave.

## Results

### Identification of the Tracemaker and Its Behavior

Specimen FOBU-12718 is interpreted as a compound swimming and feeding trail made by a bottom-dwelling teleost, specifically *Notogoneus osculus*, on the basis of interrelated qualitative and quantitative criteria, including the stratigraphic co-occurrence of the trace fossil with body fossils of *N. osculus* in the 18-inch Layer. As mentioned previously, thin, shallow impressions with regular waveforms are assignable to the ichnogenus *Undichna*; moreover, the paired, in-phase waveforms in association with a higher-amplitude waveform are best identified as *U.* cf. *simplicatas* [12], [13]. Nearly all examples of *Undichna* are ascribed to trails made by the fins of swimming fish [11], [13], [16], [17], [18], [19], [20]. Most markings of FOBU-12718 are likewise associated with fin impressions, although the medial trace is attributed to the tracemaker's mouth, explained later.

The single, high-amplitude trail is ascribed to the caudal fin, which was formed as an incision of the sedimentary surface by the ventral (distal end) of the fin as it moved the fish along the bottom. This trail is typical of a subcarangiform swimming mode, in which the posterior half of the body length propels the fish forward and most of the power stroke is derived from the caudal fin [21], [22]. The short waveform segment slightly offset from the caudal fin trace is consistent with a partial anal-fin trail. Such traces are typically lower-amplitude and partially out-of-phase waveforms in front of (and cross-cut by) caudal fin traces [13], [16], [18], [19]. The paired, parallel, and in-phase trails are interpreted as drag marks made by the distal ends of pelvic fins as the fish swam forward with an undulating motion. Pelvic fins, rather than pectoral fins, more typically form dual and relatively narrow parallel waveforms inside the higher-amplitude caudal and anal fin traces, and is more likely with a subcarangiform swimming mode, as discussed later [13], [18]. Direction of movement is also indicated by cross-cutting relations, in which the order of the waveforms (pelvic-anal-caudal) correlate with anterior-to-posterior. Hence the motion is interpretable from one end of the slab to the next, from an arbitrary left to right, as depicted in Figure 2.

**Figure 3:**
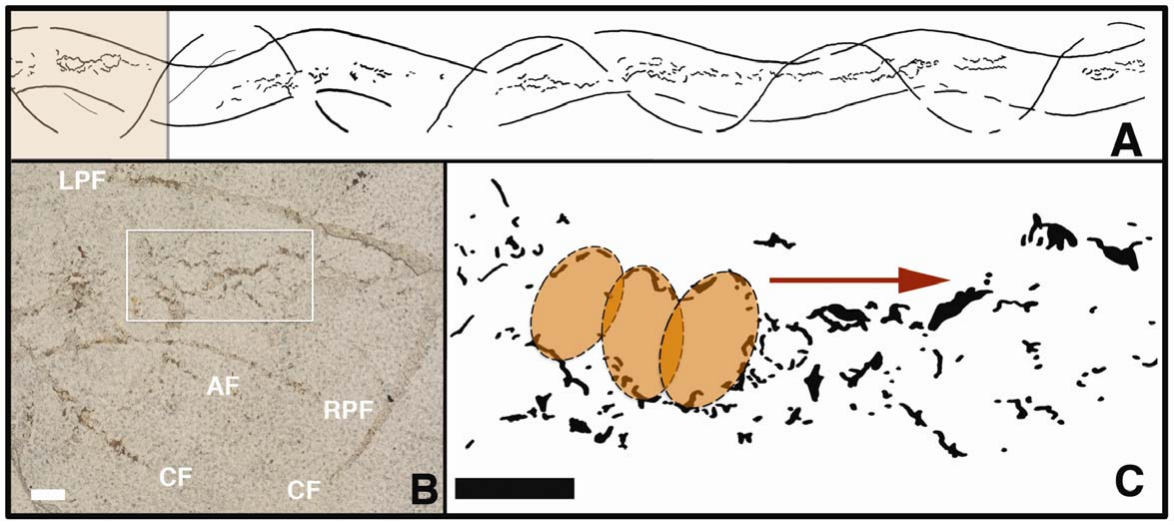
Close-up views of feeding trace in FOBU-12718. A – Overall sketch map of trace fossil, made by tracing on mylar sheet above original slab, with detailed inset (colored) for B indicated. B – Close-up of inset area in A and focus on mouth marks (box); LPF = left pelvic fin, RPF = right pelvic fin, AF = anal fin, CF = caudal fin; bar scale = 1 cm. C – Outlines of mouth traces, showing overlapping elliptical traces, based on disturbance patterns of underlying lamina; arrow indicates direction of movement in feeding; bar scale = 1 cm.

All ventrally oriented fins of the tracemaker are thus accountable as wave-like traces in FOBU-12718. As a result, a feature of the tracemaker's ventral anatomy other than its fins must have made the medial series of traces (Figure 3). We propose that the mouth is the most likely part of teleost anatomy that could have interacted with the sedimentary surface and produced such a series. Other medial parts of fish anatomy, such as claspers or additional reproductive organs associated with male chondriichthyians (e.g., myliobatiformes: [23]), would have stayed more medial to the paired, in-phase traces and in close association with the anal fin trace, rather than making the slightly undulating trace observed in FOBU-12718. Moreover, the occasional joining and bifurcating of the trace cannot be reconciled with any known clasper, nor do the other fin impressions correspond with the anatomy of any known chondriicthyian in the Fossil Lake deposit. Likewise, a simple dragging of the ventral surface of the torso would have produced a wider, more groove-like impression. Other possible tracemaking appendages on the ventral surface of a teleost are barbels, which occur in catfish. Ventrally located barbels can be maxillary (located on the sides of the mouth) or mandibular (on the “chin”), with two maxillary and four mandibular barbels as a typical arrangement. If dragged along a sedimentary bottom, barbels from a catfish-like teleost would be expected to form 4–6 intermittent and low-amplitude lineations; moreover, the most medial of such traces would be evenly spaced. However, such structures are absent. Instead, the central part of FOBU-12718 is more consistent with overlapping traces left by an oval, ring-like appendage, such as the anterior outline of the tracemaker's buccal area.

In our interpretation, the buccal diameter (medial distance between the premaxilla and dentary) is estimated as 7–12 mm (Figure 3 and high resolution image in Text S1). The anterior end of each ellipsoid then corresponds with the premaxilla impression, whereas the posterior end is from the dentary, in which “anterior” and “posterior” are defined by the interpreted direction of movement for the tracemaker (left to right in Figure 2). Of course, the continuous forward motion of the tracemaker distorted the actual outline of the mouth, and in some instances the outer edge of the trace is expressed as a zig-zag lineation with an amplitude of 7–8 mm (Figure 3 and high resolution image in Text S1). Additionally, a ventral position of the mouth, aided by a slight downward tilting of the body axis, would have placed the pelvic fins in a better position to interact with the sediment surface, while still maintaining contact of the caudal fin with the surface. Nonetheless, occasional lifting of the caudal fin off the bottom is suggested by short gaps in the caudal fin trail. Lastly, the anal fin of *N. osculus* is expected to have caused a minimal trace in comparison to that of the caudal fin, which is indeed the case in FOBU-12718.

**Figure 4:**
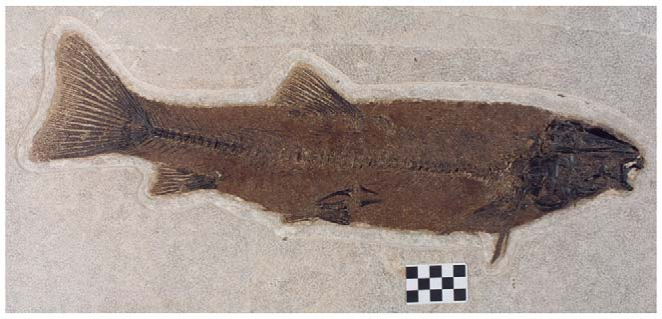
Full-size (53-cm long) adult specimen of *Notogoneus osculus* Cope, about 13% longer than the tracemaker interpreted for the trace fossil FOBU-12718; scale in centimeters. Specimen is in Fossil Butte National Monument collection; photograph by Arvid Aase.

Furthermore, we tested our identification of the tracemaker by calculating its length and comparing it to known size ranges for *N. osculus* [5]. Several researchers have proposed that fish length can be calculated on the basis on wavelength (λ_j_) or amplitude (*A*_j_) of a given fin trail [17], [21], [25], [26], [27], [28]. Using a formula derived by Bainbridge [25], and taking into account slight variation of wavelengths (λ_j_) for the pelvic and caudal impressions in FOBU-12718 (27–28 cm), estimated tracemaker length (*L*) would be 43–45 cm long (where L = λ_j_/0.62). Using formulae by Videler [21] and Wardle and others [26], estimated fish lengths are slightly less: 40–41 cm (where L = λ_j_/0.68) and 36–37.5 cm (L = λ_j_/0.75, respectively. (Formulae by Videler [21] differ slightly on the basis whether the fish was swimming in a carangiform or subcarangiform mode.) Caudal-pelvic fin distance for *N. osculus*, based on measurements taken from well-preserved fossilized specimens [5], represents on average 45.1% of the total length of the fish, and through our methods, we calculated a caudal-pelvic distance of 20.65 cm for the FOBU-12718 tracemaker. Using this ratio as a standard for comparison, and assuming *N. oscula* as the tracemaker, we thus estimated the total length of the fish as 20.65/0.451, or 45.7 cm. This length is quite close to values obtained with single-wavelength estimates [21], [25], [26]. Pooling of all four approaches results in a range of lengths from 36–45 cm (Figure 4), which is likewise close to the average total length (46 cm) estimated for *N. osculus* [1], [5]. However, given the rich potential of our methodology for refined analyses, and that we have made the raw data publicly available, we deem it as a significant and improved step towards developing a more reliable and widely applicable technique for estimating fish body sizes from their trace fossils.

Because of this evidence, in addition to the occurrence of *N. osculus* is the only known teleost species from the 18-inch Layer (and Fossil Lake) with ventrally oriented mouthparts [1], [24], we identify this species as the probable tracemaker (Figure 4). In terms of functional morphology, the only other bottom-feeding teleosts interpreted from the Green River Formation with ventrally oriented mouths include *Hypsidoris farsonensis* Lundberg and Case, *Amyzon gosiutensis* Grande et al., and *Astephus antiquus* Leidy. *H. farsonensis* is much smaller than our estimate for the FOBU-12718 tracemaker, at about 19–22 cm long, and thus far has only been found in Lake Gosiute deposits of the Green River Formation, east of Fossil Lake [1]. *A. gosiutensis* only occurs in the Laney Shale Member of the Green River Formation, having never been found in the Fossil Butte Member or Fossil Lake, and has a standard length of only 24 cm [29]. Lastly, *A. antiquus* is unknown as a body fossil in the middle unit of Fossil Butte Member, and only one specimen has been found in all of Fossil Lake, although it is abundant in Lake Gosiute strata [24]. As a result of these stratigraphic and paleogeographic disparities, we judge that *N. osculus* was the most likely tracemaker for FOBU-12718 in terms of its stratigraphic occurrence in the same stratum and location in Fossil Lake.

## Discussion

### Behavioral and Paleoecological Significance

Based on the related traits of the trace fossil FOBU-12718, *N. osculus* is identified as its tracemaker, as it is the only species known from the Green River Formation that could have contacted the sediment-water interface with its mouth while swimming with a subcarangiform motion. *N. osculus* was originally described as lacking teeth in the maxilla, premaxilla, dentary, pterygoids, or hyoids, but a reexamination of a newly found specimen confirmed teeth on the endopterygoid [5]. Regardless, this paucity of teeth in *N. osculus* led to an assumption that it fed on soft organic matter or small invertebrates along lake bottoms, perhaps through suction [1]. This interpreted functional morphology coincides with our diagnosis of the behavior for the FOBU-12718 tracemaker, in which the tracemaker was repeatedly touching the lake bottom, presumably grazing on surface algae or hunting for infaunal invertebrates. With regard to the latter possibility, we saw no evidence of invertebrate trace fossils, such as burrows or bioturbate textures, in the host lithology. Hence we are more inclined to propose that this specific tracemaker was grazing and suction feeding. Furthermore, the caudal and pelvic fins of *N. osculus* extended ventrally enough to have incised the sedimentary surface while swimming within a few centimeters of that surface, while minimizing contact of the anal fin with forward movement. In contrast, the lack of traces from the pectoral fins means these must have been elevated off the surface, and perhaps aided in swimming.

**Figure 5:**
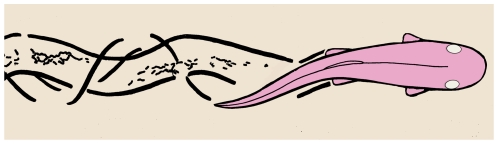
Artistic recreation of *Notogoneus osculus* forming the swimming-feeding trace fossil FOBU-12718, viewed from above.

Discontinuities of the pelvic-fin trails also likely relate to pitch and yaw of the fish while swimming. For example, a slight tilt of the axial plane of the fish to the left could have caused the right pelvic fin to lift off the surface and resulted in a gap on that side. These gaps correspond with the medial feeding trace approaching the lateral plane of the opposing side, which would have been consistent with a slight yaw as the fish swam along the lake bottom. In other words, these breaks in the continuity of the fin trails also constitute parts of the trace fossil, and have behavioral significance. Furthermore, our mathematical extrapolations of the incomplete waveforms define, with high probability, the locations of each tracemaking appendage above the sedimentary surface, even where no traces were made, as well as the size of the fish (Figure 5).

Besides the first known linkage in the geologic record of a trace fossil with *N. osculus*, the most significant implication of this discovery is of this teleost behaving normally in the deepest-water portion of Fossil Lake. Middle Unit deposition took place during a maximum high-stand (transgression) of Fossil Lake, which is associated with wetter climatic conditions [6]. The relatively high (2–14%) total organic contents (TOC) of the kerogen-rich laminated micrites (KRLMs), which compose the 18-inch Layer and much of the remaining Middle Unit in the Fossil Butte Member, has been attributed anoxic to dysaerobic depositional environments [1], [3], [4]. Moreover, the lack of infaunal bioturbation in KRLMs, which grade laterally into nearshore bioturbated mudstones, supports that these beds are indeed closer to the depositional center of Fossil Lake [3], [4]. This paleoenvironmental interpretation has been applied specifically to the 18-inch Layer, which is reinforced by the paucity of clastic sediment (less than 5%) composing this stratum, as well as excellent preservation of teleost body fossils serving as indicators of distance from shore [1], [2], [4].

As mentioned previously, *N. osculus*, with its ventrally oriented mouth, was likely adapted for benthic feeding. Nonetheless, this feeding was originally assumed to have taken place in shallow-water environments of Fossil Lake or in nearby freshwater streams feeding into the lake [1], [5], despite the rarity of its body fossils in shallow-water strata [24]. This explanation also partially accounts for its relative scarcity compared to other nekton in the 18-inch Layer, which is about 3% of all fish taxa [24]. As a result, it may have only swum into the central (deeper) parts of the lake during seasonal turnovers that altered stratified (meromictic) conditions [1], [5]. Because *N. osculus* was presumed to have fed on bottoms under shallower depths than those interpreted for the 18-Inch Layer, most of its body fossils, accordingly, can be considered as allochthonous. This presumption, though, is contradicted in at least one instance by the trace fossil described here, which may indeed represent a time of at least temporary oxygenation of bottom waters. As a result, FOBU-12718 and other specimens of *Undichna* from the 18-inch Layer could represent seasonally linked behavior of Fossil Lake teleosts, although this idea requires more testing. Unfortunately, extant gonorynchid relatives of *N. osculus*, such as various species of *Gonorynchus*, provide imperfect models for seasonal variations in behavior, as these are all exclusively marine and live in the Indo-Pacific region. Nonetheless, their similar anatomies and bottom-feeding habits in shallow-marine environments, the latter lending to their nicknames as “sand fish” [5], might provide actualistic models of their swimming and feeding patterns, which can be compared to our interpretations.

Fish coprolites had been identified previously from the Green River Formation [30] and only one trace fossil interpreted as a fish trail (*Undichna*) was previously reported from the Fossil Lake deposit [31], hinting at further finds of such trace fossils. Indeed, other specimens of *Undichna* attributable to teleosts have been found since then in the 18-Inch Layer and other fish-bearing zones of the Green River Formation in the area of Fossil Butte, which will be the subjects of future study (Table 2). In the meantime, these teleost trace fossils confirm that other fish were also swimming along the sediment-water interface of Fossil Lake during Middle Unit time. As a result, FOBU-12718 and other specimens of *Undichna* may be only a few of many more examples of teleost trace fossils from this bed.

**Table 2:** Additional *Undichna* specimens from the 18-inch Layer, Middle Unit, Fossil Butte Member; ranges of amplitudes and wavelengths for interpreted caudal-fin trace of each specimen; and ichnospecies of each specimen, with ichnotaxonomy based on that of Minter and Braddy [13].

Specimen	Amplitude	Waveform Length	Ichnospecies
FOBU-3145	6.5–7	9–10	*U. unisulca*
FOBU-11709	9.5–10	25	*U. trisulcata*
FOBU-11710	8–10	14–16	*U. simplicitas*
FOBU-11711	8–9	30–32	*U. simplicitas* or *U. quina*

This prediction about teleost trace fossils in Fossil Lake deposits will be examined critically as more people working on the Green River Formation, who have been trained mostly to look for body fossils, are made aware of this potentially rich source of scientific information represented by teleost trace fossils. Teleost trace fossils have certainly contributed to paleoecological interpretations in other studies of ancient lacustrine deposits [32], [33]. Consequently, we hope the results of our study will prompt a similar expansion of paleoecological insights relating to Green River strata in and around Fossil Butte National Monument.

### Conclusions

The Green River Formation is a deposit world-famous for its fossil teleosts, but relatively little work had been conducted previously on its fish trace fossils. This paucity of ichnological data is advanced considerably by FOBU-12718, an extraordinary trace fossil from the 18-inch Layer of the Fossil Lake Member (Green River Formation, early Eocene) in Wyoming. This combined swimming and feeding trace fossil provides the first known independent evidence of bottom-feeding behavior in *Notogoneus osculus* Cope (Teleostei: Gonorynchidae), a behavior that had been previously interpreted for this species [1], [5]. Our study was also the first to apply digital spatial-analysis tools and Fourier series to the description and interpretation of a teleost trace fossil, providing alternative methods for estimations of fish size and motion.

The ventrally oriented mouthparts of *N. osculus*, rare among fossil teleosts from the Green River Formation, comprised the only previous evidence of a bottom-feeding habit in this species. The trace fossil is from the same stratum containing the only known specimens of *N. osculus* from this area, further narrowing the identity of the tracemaker. This trace fossil and other swimming traces from the same stratum refute suggestions that benthic conditions in Fossil Lake of the Green River Formation were permanently anoxic and inhospitable for bottom-dwelling teleosts. Our discovery thus prompts a reexamination of the previously interpreted paleoecology of Fossil Lake, and adds bottom feeding as an aspect of the benthic ecology in the deepest part of the lake.

The trace fossil represented by specimen FOBU-12718 is indeed special in showing more detailed evidence of behavior other than a teleost simply swimming along a lake bottom. This trace fossil also demonstrates the utility of ichnology in testing previous interpretations of teleost behavior based on functional morphology, while refining paleoecological interpretations of Green River teleosts in conjunction with their abundant and well-preserved body fossils in Fossil Lake. This discovery also suggests that, at least for brief periods, lake bottom waters were occasionally oxygenated and provided feeding opportunities for near-benthic fish, such as *Notogoneus osculus*, rather than excluding them, as was previously supposed.

Lastly, the trace fossil itself represents only a fleeting moment in the tracemaker's interactions with the lake bottom, but has broad implications in terms of interpreting the paleoecosystems of Fossil Lake. This snapshot of teleost behavior from Fossil Lake is thus all the more remarkable for its brevity and apparent rarity, but also for how it potentially alters previous assumptions about the paleoecology of Fossil Lake.

## Supporting Information

Text S1 Supplementary text.
(0.07 MB DOC)

Figure S1 Cross-correlogram between caudal and pelvic fin distances derived for FOBU-12718. Lags are represented in cm from the caudal fin.
(0.08 MB TIF)

File S1 This datafile contains the raw XY coordinates (in mm) from all the FOBU-12718 identified appendages and mouth marks.
(0.08 MB XLS)
