# Supplementary material for: First Known Feeding Trace of the Eocene Bottom-Dwelling Fish Notogoneus osculus and Its Paleontological Significance
Source: PLoS One. 2010 May 5;5(5):e10420. doi: 10.1371/journal.pone.0010420 (PMC2864752; doi:10.1371/journal.pone.0010420)
Supplement: Text S1 — Supplementary text. (0.07 MB DOC) [file pone.0010420.s001.doc]

**Text S1**

**First Known Feeding Trace of the Eocene Bottom-Dwelling Fish *Notogoneus osculus* and its Paleontological Significance**

Anthony J. Martin, Gonzalo M. Vazquez-Prokopec, Michael C. Page

Department of Environmental Studies, Emory University, Atlanta, GA 30322, United States of America

**SUPPLEMENTAL MATERIALS**

**Utilization of Microsoft’s Seadragon Technology in Exploring an Eocene Fish Trace Fossil**

The display of high-resolution images has always been problematic on computer monitors. With Microsoft’s recent development of Seadragon™ software, users are now able to view such images through a Web browser, making use of Microsoft Silverlight™ technology. This software allows users to fluidly pan across and zoom in on a large, high-resolution image by only displaying the viewed region at full resolution. Adjacent regions of the viewing area are downloaded to the client, with the user viewing a specific region, and animations are used to create smooth transitions when the user pans or zooms to another region. This technology greatly improves the examination of large high-resolution images distributed over the Internet and viewed through a Web browser.

For the specimen examined in this study, FOBU-12718 from the Green River Formation near Fossil Butte National Monument (Wyoming, USA), this new technology allows us to effectively present visual details of this remarkable trace fossil to the general public, while also allowing for an interactive image. The image was made through stitching a series of high-resolution digital photographs using Adobe Photoshop (version CS3) and is the same image that was spatially referenced using ArcGIS 9.2 for the analysis described in our article. The stitched image was processed in Microsoft’s Deep Zoom Composer™, which divides the images into tiles, then builds an image pyramid of images at tiered resolutions. This process is very similar to current techniques used in remote-sensing applications and geographic information systems (GIS) when handling raster data for display. Although the resulting Deep Zoom image is not spatially referenced, the user is able to explore the image via the pan-and-zoom interface.

To begin viewing the Deep Zoom image, the user needs to go to the following URL:

<http://edc.library.emory.edu/datalib/eocene/DeepZoomProjectWeb/ClientBin/fistrace.html>

Once at the site, the user can download the free Microsoft Silverlight™ plug-in and install it on her or his Web browser if needed. Once installed, the user should return to the URL, where the image should be viewable in Deep Zoom mode. Through this software, the user will be able to visually inspect nearly all visible features of the trace fossil, and in extreme detail.

For editorial and archival purposes, the content for this page is permanently at:

<http://pid.emory.edu/ark:/25593/7qpdd>

**Estimation of Distance between Caudal and Pelvic Fins: Further Explanation**

A subcarangiform swimming mode is characterized by an undulating movement of the distal portion of the fish that extends from caudal to pelvic fins. This fish movement was taken into consideration when estimating the distance between appendages (i.e., between caudal and pelvic fins) for the tracemaker of FOBU-12718. We calculated the distance separating peaks and valleys between subsequent caudal and pelvic waveforms (see Figure 5) by applying a cross-correlation function [1]. Briefly, we first estimated the central pelvic wave (representing the median body axis) by averaging the upper and lower pelvic fin traces. We then estimated the value of the fitted caudal and central pelvic waveforms over the range 0-108 cm with a resolution of 0.1 cm using Matlab 10.1 curve-fitting tool. A cross-correlation function was applied between such fitted waveforms, and the cross-correlation product (*r*) plotted against the lag distance (measured in cm from the caudal fin), as shown in Figure S1. The cross-correlation between caudal and pelvic waveforms was positive at 7.5 cm (range, 6.3-7.9 cm) from the caudal fin, and negative at 20.65 cm (range, 19.1-22.6 cm). The latter measure represents the distance between a peak in the caudal fin and a valley in the pelvic central fin, and can be interpreted as the distance between such appendages (as represented in Figure 5). Cross-correlations were performed using STATA 10.1 software (STATA Corp., College Station, TX).

**Reference**

1. Shumway RH, Stoffer DS (2006) Time series analysis and its applications: with R examples. New York: Springer. xiii, 575 p.
