# Supplementary figures and images for: First Known Feeding Trace of the Eocene Bottom-Dwelling Fish Notogoneus osculus and Its Paleontological Significance
Source: PLoS One. 2010 May 5;5(5):e10420. doi: 10.1371/journal.pone.0010420 (PMC2864752; doi:10.1371/journal.pone.0010420)

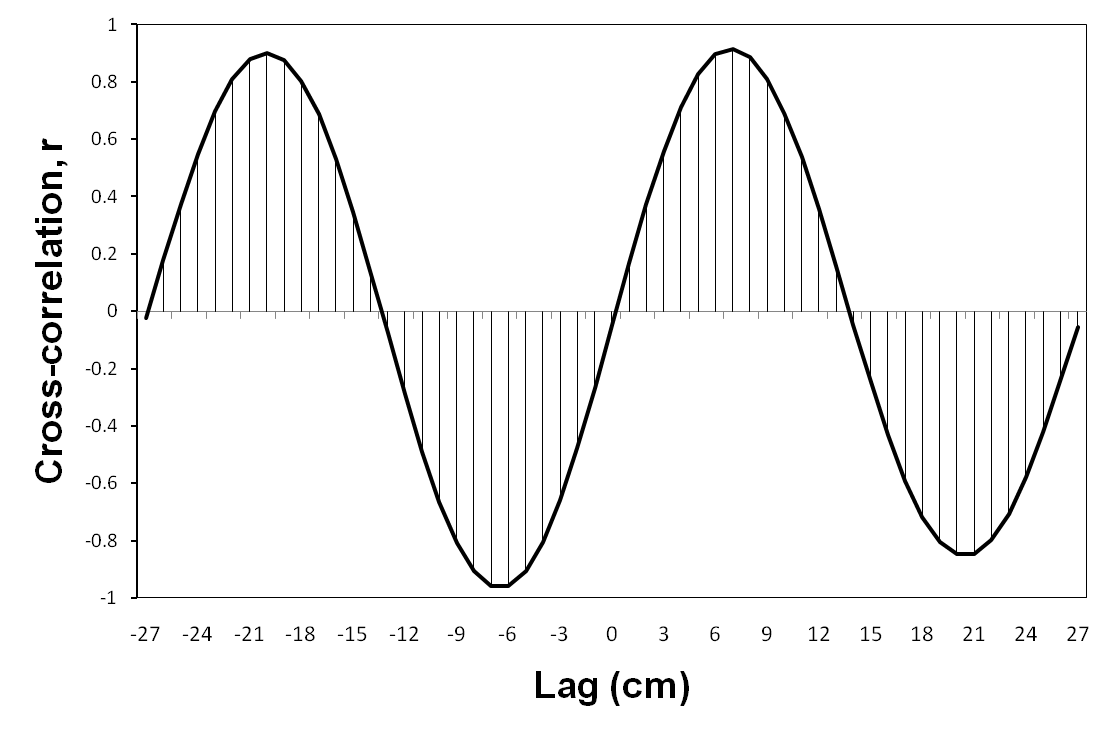

Supplement: Figure S1 — Cross-correlogram between caudal and pelvic fin distances derived for FOBU-12718. Lags are represented in cm from the caudal fin. (0.08 MB TIF) [file pone.0010420.s002.tif]
